# Comparing the Effects of Melatonin and Zolpidem on Mental Health and Sexual Function in Men With Opioid Addiction: Evidence From a Randomized Clinical Trial

**DOI:** 10.3389/fpsyt.2022.850480

**Published:** 2022-02-28

**Authors:** Zahra Amini, Mina Moeini, Negin Etminani

**Affiliations:** ^1^Department of Community and Family Medicine, School of Medicine, Isfahan University of Medical Sciences, Isfahan, Iran; ^2^Vice-Chancellor of Health Affairs, Isfahan University of Medical Sciences, Isfahan, Iran; ^3^Department of Community Medicine, Isfahan University of Medical Sciences, Isfahan, Iran

**Keywords:** melatonin, zolpidem, sexual function, mental health, methadone maintenance therapy

## Abstract

**Background:**

Mental health problems and impaired sexual function are widely reported among those suffering from drug abuse, particularly among those under methadone maintenance therapy (MMT).

**Aims:**

The current study aimed to, firstly, investigate the effect of melatonin and zolpidem on mental health and sexual function of those with drug abuse under MMT, and, secondly, to compare the effects of melatonin and zolpidem on the studied outcomes.

**Methods:**

The current randomized, single-blind, placebo-controlled clinical trial was conducted on 98 participants who were randomly assigned into three groups of melatonin (*n* = 34), zolpidem (*n* = 32), and placebo (*n* = 32). All participants received the intervention once a day for 30 days, without changes in nutrition. Mental health and sexual function were measured before and 30 days after the intervention.

**Results:**

The mean age of participants in the groups of melatonin, zolpidem, and placebo was 35.8 ± 9.6 years (22–58 years of old), 35.9 ± 9.3 years (21–58), and 37.2 ± 7.8 years (26–53), respectively. Sexual function mean score was significantly increased from 38 to 41 in the melatonin group, while it deceased in zolpidem (from 39.1 to 38) and placebo (39.25–38.59) groups. Also, mental health mean scores improved statistically significantly in the melatonin group (from 60.65 to 43.56; *p* = 0.002), and descriptively in the zolpidem group (57.88–51.18; *p* = 0.129). Concerning both outcomes, the observed improvement was considerably higher in the melatonin group. The highest improvement was observed in dimensions of overall satisfaction and depression in the melatonin group (1.18 and −8.4, respectively).

**Conclusion:**

Melatonin could significantly improve both mental health and some domains of sexual function of those with drug abuse under MMT, while zolpidem did not show a significant effect.

**Trial Registration Number:**

https://www.irct.ir/trial/53047, identifier: IRCT20201214049718N1.

## Introduction

In today's modern world, inclination toward opioid addiction is on the rise, due to several reasons such as stress from working long hours, high costs of living, etc. (1–3), not to mention the effects of the Covid-19 pandemic (4). The United Nations reported that about 275 million people used opioid combination products worldwide in 2020, with a growth rate of 22% from 2010 (5). In addition, there are evidence indicating that mental health disorders and opioid addiction co-exist in most cases, as in response to mental problems some will try to address them with opioid combination products (6, 7). So that nearly 50% of those who suffer from mental disorders are affected by opioid addiction (8). In addition, opioid addiction may magnify many mental health problems (9), which in turn enhances inclination toward more addiction (10, 11). Also, one of the most common complaints of those who are trying to quit opioid addiction is mental health problems (12, 13). Furthermore, methadone maintenance therapy (MMT), which is widely used to treat opioid addiction by preventing opioid withdrawal and reducing cravings, is associated with increased risk of worsened mental health problems (14). It is worth noting that addicted persons suffering from psychological health problems are at increased risk of having lower quality of life (QoL) (15), which in turn translates into higher mortality rates (14).

Furthermore, those with opioid addiction also suffer from impaired sexual function (16). While such dysfunctions may occur in any stage of the normal sexual cycle, erectile dysfunction (ED) or impaired orgasm function are among the earliest issues (17), which cause an extra burden, with long-term consequences, on their mental health problems, apart from the dissatisfaction of their partners. Therefore, evaluating the mental health and sexual function of those who suffer from opioid addiction can provide valuable information about how to address their problems more efficiently, which in turn paves the way for better and faster quit.

Currently, several types of interventions are available for this purpose, including pharmacological, behavioral, cognitive, and hormonal. Pharmacological interventions (e.g., benzodiazepine, bupropion, trazodone) are one of the most efficient interventions with immediate effects; however, caution should be taken as often lead to addiction or cause side effects, which results in more declines in the QoL (18). Herbal medicines such as Ginseng and Rosa Damascena Oil have also been studied, but drug interactions, the time required for effectiveness, and the acceptance of patients in the use of herbal medicines should be considered (19, 20). While the impacts of other interventions would be observable in the long term. There are extensive evidence regarding the potential effects of melatonin on reducing inclination toward opioid addiction and relapse of addiction, as well as regulation of mental health mechanisms (21). In addition, melatonin is associated with improved mental health, through alleviating lipopolysaccharide-induced anxiety, which indicates its therapeutic effect (22, 23). The pineal gland secrets melatonin, and its secretion decreases during periods of depression, which is another reason to support its unique role in mental health regulation. In addition, zolpidem is one of the most effective non-benzodiazepine medications available to treat mental health issues, mainly through activating GABA receptors, which in turn opens chloride channels, declines the firing rate of neurons and muscle fibers, and selectively binds to the subunit-specific GABA receptors (24).

In this study, we sought to use a drug in addition to methadone that could address common patient problems such as mental and sexual problems. Usually, people on methadone treatment suffer from these problems and increase the dose of their methadone drug to solve it. We were looking for a drug that we could use to manage several common complaints of these patients. To the best of our knowledge, few studies have simultaneously investigated the effects of melatonin and zolpidem supplementation on mental health and sexual function of those under MMT. “We expected melatonin to be more effective than zolpidem and placebo.” Based on what was mentioned before, the purpose of this article is to, firstly, investigate the effect of melatonin and zolpidem on depression, anxiety and stress dimensions of mental health and erectile function aspect of sexual function of those under MMT, and, secondly, to compare the effects of melatonin and zolpidem on the studied outcomes.

## Methods

### Study Design and Participants

The current randomized, single-blinded, placebo-controlled clinical trial is carried out on males receiving outpatient treatment for opioid addiction in the city of Isfahan, Iran in 2021. Participants were selected from a single center. All eligible participants were followed up for 1 month (from April 3, 2021, to May 22, 2021), and to prevent contamination, they were asked to refer on particular days.

The sample size was estimated as 32 subjects per group, following the study by (16, 21) with a 95% confidence interval and 80% statistical test power, using the following formula:


$$\text{n}=2^{*}(\text{z}\_(1-\alpha /2)+\text{z}\_(1-\beta ))2\times \text{sd2})/\text{d2}$$

However, allowing for a 10% dropout, the estimated sample size per group was increased to 35. Consequently, a total of 105 subjects with opioid addiction under the MMT with sleep quality of at least 5 were selected using convenience sampling. Then, using the Excel software they were divided into three groups of melatonin, zolpidem, and placebo. Those with the following criteria were included: (1) older than 18 years of old; (2) ability to read and write; (3) Opioid addiction, confirmed by urine test; (4) a Pittsburgh sleep quality index score more than 5; (6) No history of neurological diseases, neuro psychosis disorder, autoimmune diseases, cancer, lung disease, heart failure class 4 or unstable angina; (7) No history of receiving benzodiazepine, anticonvulsant, aspirin, beta-blockers, calcium channel antagonists, NSAID, dexamethasone, lithium, antidepressants such as serotonin and melatonin reabsorption inhibitors; (8) Not working at night shifts; (9) Receiving MMT at least for three months; and (10) Willingness to participants. Participants were excluded if were: (1) Unwilling to continue the study; (2) Experienced changed treatment protocol; (3) History of sensitivity to either melatonin or zolpidem; and (4) pregnant or breastfeeding.

### Data Collection

Data were collected using a demographic questionnaire, as well as Anxiety, and Stress Scale-21 Items (DASS21), and International Index of Erectile Function (IIEF).

### Depression, Anxiety and Stress Scale-21 Items

The DASS21 is a set of three self-report scales intended to measure states of depression, anxiety, and stress. Participants should score each item on a four-point Likert scale, ranging from zero (“did not apply to me at all”) to three (“applied to me very much”). To obtain the total score, the scores obtained for each dimension should be summed up and then multiplied by a factor of 2. The total score ranges from zero to 120 and those for each subscale ranges from zero to 42 (25). The Persian version of the DASS21 is evaluated by NikAzin et al. and a Cronbach alpha of 0.7, 0.84, and 0.82 is reported for dimensions of depression, anxiety, and stress, respectively.

### International Index of Erectile Function

Developed by Rosen and Cappelleri (26), the IIEF is a widely used scale to evaluate male sexual function. As a self-administered questionnaire, the IIEF contains 15 items that are categorized into five dimensions of erectile dysfunction, orgasm, desire, satisfaction with intercourse, and overall satisfaction. It should be noted that the first dimension is reverse scored. Hence, for other dimensions, higher scores indicate better functioning (26). Rajabi et al. (27) reported a Cronbach alpha of 0.91 for the Persian version of this scale (27).

### Randomization

Initially, 123 potential subjects were proposed to participate, in which 105 of them accepted our invitation. Then, 105 participants were randomly allocated to one of the melatonin, zolpidem, and Placebo groups using random selection from a list prepared in Excel software. The latter was considered as control, and the rest were defined as intervention.

### Intervention

**Figure 1:**
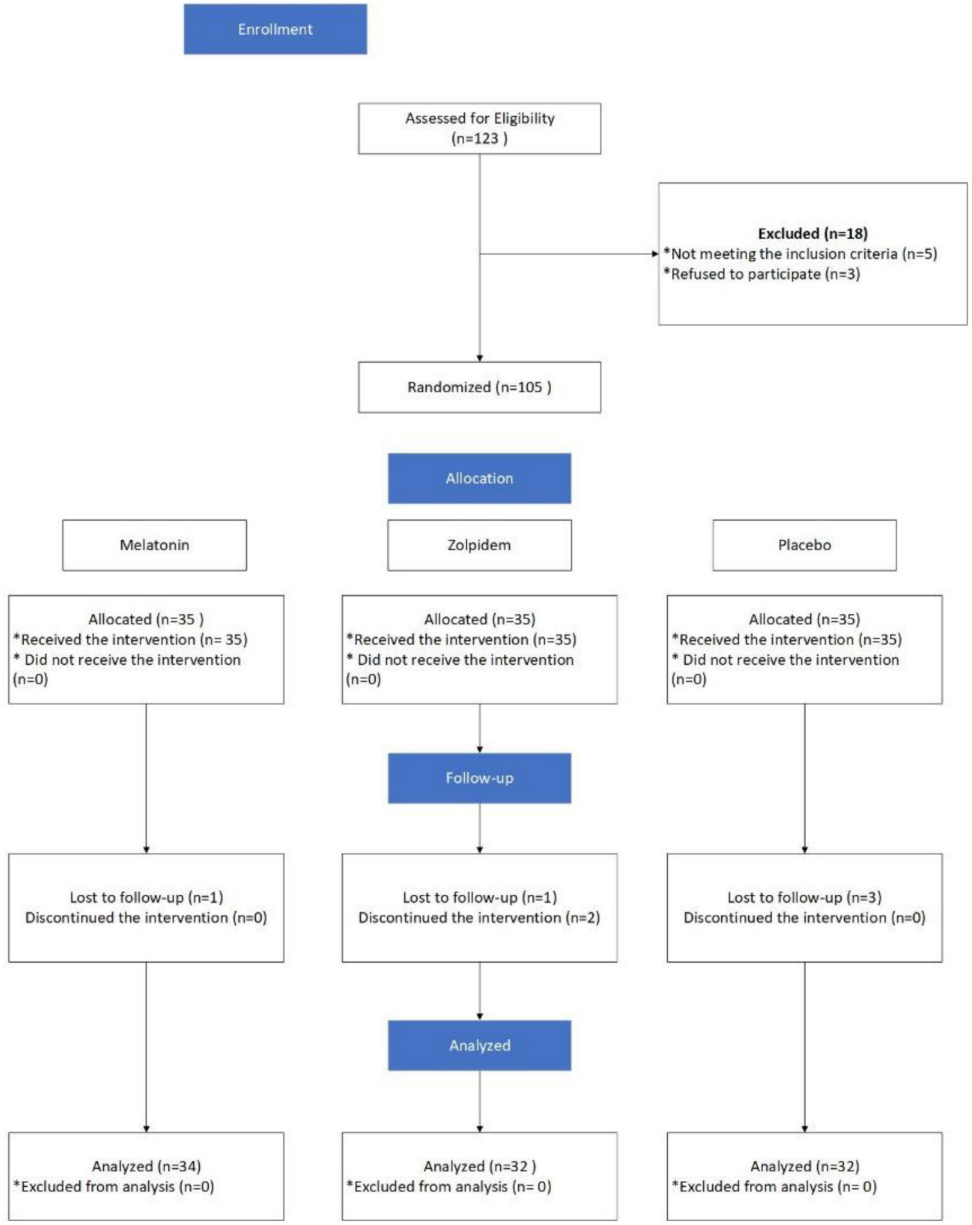
Allocation process of the study.

Subjects in the zolpidem group were provided with 10 mg tablets (Manufactured by Exir Pharmaceutical Company, Boroujerd City, Iran). Participants of this group were provided with 7 tablets at the beginning of the study and were asked to refer each week for receiving new tables. In addition, they were asked take one tablet per night at bedtime. Those in the melatonin group, as MMT decreases response to routine doses of sleeping pills, were asked to take three 3 mg tablets (Razak melatonin, Karaj, Iran 3 mg = 9 mg) 30 min before sleep. Initially, a total of 21 tablets were provided to them. Those in the placebo group were given placebo tablets, made of Starch, similar to tablets in the intervention groups in shape and color (produced by a pharmacist living in the Isfahan city). The prescription dose of melatonin and zolpidem was determined based on the best efficacy of the medicines according to previous experiences of the research team. The interventions lasted for 30 days, and each participant in the zolpidem and control groups used a total of 30 tablets, while those in the melatonin group received 90 tablets. All participants were asked to refer to the center to fill the data collection tools after 30 days. In addition, they were asked to return tablets that they did not take. It is worth noting that all participants were asked to consume a regular diet and sleep in completely dark rooms to control the potential effects of nutrition status and nighttime lighting on serum levels of melatonin. Participants filled the study questionnaires two times (before and 30 days after initiating the intervention). To reduce the attrition rate, all participants were reminded of the importance of taking tablets when referring to receive in one's weekly. Furthermore, the tablets were given to participants by one member of the research team and others were unaware of the type of medicines taken by participants. The groups' allocation, interventions, and follow-up, and the analysis of the results are indicated in Figure 1.

### Statistical Analysis

Descriptive statistics were used to summarize the collected data. The Kolmogorov-Smirnov test was applied to test for a normal distribution, which indicated a normal distribution for all variables. In addition, paired *t*-test and analysis of variance (ANOVA) were used for between-group comparisons regarding baseline variables. Partial eta squared was used to show the effect size for the ANOVAs. Bonferroni *post-hoc* test was used to compare two groups. The chi-square test was used to compare the study groups regarding the variable of occupation, marriage status, and smoking. In addition, the Kruskal-Wallis H test was used to compare the study groups regarding various levels of education. Data analysis was administered using SPSS version 23. Statistical significance was considered when *p* < 0.05. The intention to treat approach was applied.

## Result

Initially, 105 participants were recruited, in which data of 98 of them were available for final analysis (34 in the melatonin, 32 in the zolpidem, and 32 in the placebo groups). Seven subjects were lost due to loss to follow-up, failure to receive the intervention, or reporting side effect (Figure 1). The youngest and oldest participants were 21 and 58 years old. The mean age of participants in the groups of melatonin, zolpidem, and placebo was 35.8±9.6 years (22 to 58 years of old), 35.9 ± 9.3 years (21 to 58), and 37.2 ± 7.8 years (26 to 53), respectively. No significant differences were observed for age and methadone dose in three groups. According to the chi-square test, there was no significant difference between the study groups concerning the variables of occupation (*p* = 0.28) and marital status (*p* = 0.54). In addition, the chi-square test with the likelihood ratio showed no significant difference between the three groups concerning the smoking status (*P* = 0.93; Table 1). The frequency distribution of various levels of education is also provided in Table 1. According to the results of the Kruskal Wallis H test, there was no significant difference between the study groups concerning the variable of education (*P* = 0.72). In addition, most of the participants had the ability to read and write (*n* = 34; 34%) or a degree of less than diploma (*n* = 34; 34%). There was no significant difference between the study groups concerning the variable of age, according to the one-way ANOVA test (*P* = 0.79).

**Table 1:** Comparison of demographic characteristics in the study population.

**Characteristics**		**Melatonin**	**Zolpidem**	**Placebo**	**Test statistics^ł^**	***P*-value**
Age (year), Mean ± SD^1^		35.8 ± 9.6	35.9 ± 9.3	37.2 ± 7.8	0.233	0.79^a^
Methadone dose (mg)		17.3 ± 66.18	17.32 ± 62.88	16.32 ± 70.47	1.628	0.20^a^
Marital status	Married (%)	14 (41.2)	12 (35.5)	9 (28.1)	1.236	0.54^b^
	Single (%)	20 (58.8)	22 (64.7)	23 (71.9)		
Education level	Ability to read and write	14 (41.2)	11 (32.4)	8 (25)	0.652	0.72^c^
	Less than Diploma	14 (41.2)	17 (50)	19 (59.4)		
	Diploma	2 (5.9)	5 (14.7)	4 (12.5)		
	Bachelor	2 (5.9)	1 (2.9)	1(3.1)		
	Master's degree	2 (5.9)	0	0		
Occupation status	Employed	18 (57.9)	12 (35.3)	12 (37.5)	2.564	0.28^b^
	Unemployed	16 (47.1)	22 (64.7)	20 (62.5)		
Smoking	Yes	29 (85.3)	30 (88.2)	28 (87.5)	0.139	0.93^b^
	No	5 (14.7)	4 (11.8)	4 (12.5)		

a *ANOVA test*,

b *Chi-Square test*;

c *Kruskal Wallis H test*.

1 *Standard deviation*.

ł *Test Statistics for ANOVA: F, Test value for Chi-square test: Pearson Chi Square, Test value for Kruskal Wallis H test: Chi-square*.

Mean scores of sexual function and mental health status both before and1 month after the intervention are provided in Tables 2, 3. Concerning the sexual function, those who received melatonin experienced improved sexual function (+2.56; *p* = 0.035), while subjects of zolpidem and control groups experienced reduced mean scores (−1.17 and −0.66, respectively; *p* = 0.335 vs. 0.576), which means worsened function (Table 2). Those in the melatonin group experienced improved scores in dimensions of sexual desire (*p* < 0.0001), and overall satisfaction (*p* = 0.001), which was not statistically significant for intercourse satisfaction. Meanwhile, the orgasmic function and erectile dysfunction did not statically change (*p* = 0.998, 0.514). On the other hand, sexual desire (*p* = 0.034) were improved for participants in the zolpidem group. Also, the ED was declined, which indicates an improvement (−0.97; *p* = 0.108), which was not statistically significant. In addition, orgasmic function (*p* = 0.456) and intercourse satisfaction (*p* = 0.203) were reduced, while not being statistically significant. However, the reported effect size of the interventions (melatonin and zolpidem) for sexual function were small 1 month after the intervention.

**Table 2:** Comparison of changes in the scores for sexual function domains in the three groups.

**Study variables**		**Before the intervention** **M ±SD**	**One-month after** **M ±SD**	**P^**1**^**	**P^**2**^**	**Partial eta square^$^**
Erectile dysfunction	Melatonin	15.17 ± 6.82	14.76 ± 8.16	0.514	0.443	0.005
	Zolpidem	15.35 ± 7.32	14.38 ± 8.67	0.108		
	Control	14.78 ± 7.75	13.28 ± 8.85	0.014^*^		
Orgasmic function	Melatonin	5.82 ± 1.73	5.82 ± 1.84	0.998	0.782	0.004
	Zolpidem	5.73 ± 1.91	5.52 ± 2.10	0.456		
	Control	5.93 ± 1.74	5.71 ± 1.90	0.325		
Sexual desire	Melatonin	5.52 ± 1.65	6.64 ± 1.64	<0.0001^*^	0.779	0.005
	Zolpidem	5.88 ± 1.62	6.401 ± 1.79	0.034^*^		
	Control	6.15 ± 1.54	6.37 ± 1.68	0.415		
Intercourse satisfaction	Melatonin	6.76 ± 3.07	7.44 ± 3.98	0.191	0.273	0.026
	Zolpidem	6.91 ± 3.16	6.32 ± 3.96	0.203^*^		
	Control	7.09 ± 3.76	5.93 ± 3.82	0.023^*^		
Overall satisfaction	Melatonin	5.32 ± 2.51	6.50 ± 2.04	0.001^*^	0.066	0.055
	Zolpidem	5.29 ± 2.69	5.35 ± 2.46	0.863		
	Control	5.28 ± 2.72	5.34 ± 2.39	0.837		
Total	Melatonin	38.61 ± 13.38	41.17 ± 14.51	0.035^*^	0.672	0.008
	Zolpidem	39.17 ± 14.69	38.00 ± 16.47	0.335		
	Control	39.25 ± 15.46	38.59 ± 15.64	0.576		

*P^1^: Paired T-Test at 5% level; P^2^: At the 5% level of multivariate ANOVA*.

$ *Partial Eta Square interpret: 0.01 ~ small, 0.06 ~ medium, >0.14 ~ large*.

* *Statically significant p value*.

**Table 3:** Comparison of changes in the scores for mental health domains in the three groups.

**Study variables**		**Before the intervention** **M ±SD**	**One-month after** **M ±SD**	**P^**1**^**	**P^**2**^**	**Partial eta squared^$^**
Depression	Melatonin	20.9 ± 9.7	12.5 ± 6.9	<0.0001	0.0001	0.197
	Zolpidem	20.2 ± 6.7	17.9 ± 7	0.120		
	Control	20.1 ± 7.5	20.7 ± 7.03	0.668		
Anxiety	Melatonin	18.9 ± 8.5	15.6 ± 8.3	0.103	0.06	0.056
	Zolpidem	18 ± 5.8	15.4 ± 6.6	0.089		
	Control	19.4 ± 6.4	19.1 ± 5.4	0.789		
Stress	Melatonin	20.8± 8.9	15.5 ± 8.2	0.012	0.019	0.078
	Zolpidem	19.7 ± 5.7	17.9 ± 7.9	0.340		
	Control	20.5 ± 7.3	20.8 ± 6.2	0.842		
Total	Melatonin	60.65 ± 24.12	43.56 ± 19.42	0.002	0.025	0.128
	Zolpidem	57.88 ± 16.08	51.18 ± 19.30	0.129		
	Control	60.00 ± 18.97	60.56 ± 15.78	0.886		

*P^1^: Paired T-Test at 5% level; P^2^: At the 5% level of multivariate ANOVA*.

$ *Partial Eta Square interpret: 0.01 ~ small, 0.06 ~ medium, >0.14 ~ large*.

For those who received the placebo, while the sexual function is declined, for dimensions of intercourse satisfaction (i.e., −1.16; *p* = 0.023), the only statistically significant improvement was related to the ED dimension (*p* = 0.014).

The results of paired *t*-test and ANOVA tests concerning the impact of the interventions on depression, anxiety and stress aspects of mental health are provided in Table 3. According to the findings, both interventions could significantly improve the mental health of participants (*p* = 0.025); however, only those related to the melatonin were statistically significant. Also, those in the melatonin group experienced a considerably higher improvement compared to the zolpidem group (−17.9 vs. −6.7; *p* = 0.002 and *p* = 0.129, respectively). Meanwhile, those in the placebo group experienced a slight increase in their mental health score, which indicates worsened mental health status (i.e., 0.56; *P* = 0.886), which were not statistically significant.

Concerning three dimensions of mental health (i.e., depression, anxiety, and stress), those in the melatonin group had improved scores; which was significant for depression (*p* < 0.0001) and stress (*p* = 0.012). In the same vein, subjects of the zolpidem group also had a reduced mean score, which means better health status, for all dimensions. Nevertheless, the observed improvement was not statistically significant for none of them. On the other hand, only the anxiety was slightly improved among those in the placebo group (−0.3; *p* = 0.789). Bonferroni *post-hoc* test showed that those in the melatonin group experienced more improvement compared to the other two groups in terms of depression (*p* = 0.006), while the zolpidem and control groups did not differ significantly (*p* = 0.317). In terms of anxiety, the three groups did not differ significantly. For stress dimension of the mental health; the mean score in the zolpidem group was not significantly different from the other two groups (*p* = 0.587, *p* = 0.353), but the melatonin group was significantly better than the control group (*p* = 0.015). so that an improvement equal to (−17.09) was observed in the melatonin group compared to (−6.7) in the zolpidem group (*p* = 0.002 vs. *p* = 0.129). Melatonin had the greatest effect on the dimension of depression, which had the highest effect size) partial eta squared = 0.197).

## Discussion

There are evidence indicating the negative impacts of both opioid addiction and MMT on mental health (28) and sexual function (17). In the present study, we investigated the impact of melatonin and zolpidem on these outcomes, and a comparison is provided concerning their effects, using a placebo group.

Sexual dysfunction is defined as psychophysiological changes that affect the sexual response cycle and cause impaired sexual desire (29). The association between depression and declined sexual function is well-established, particularly through decreased libido and ED (30). There are evidence indicating that melatonin can improve sexual function by declining the arousal threshold by moderating the sensitivity of the central 5-hydroxytryptaminergic receptor (31). However, it should be considered that various factors contribute to sexual function, including sex, education, depression, and socio-economic status (32), in which some of them are controlled in the present study. Other possible reasons for the positive impacts of melatonin on sexual function are reduced oxidative stress and preventing cell apoptosis in the central nervous system.

The results of the present study showed that melatonin could significantly improve the mean score of sexual function and mental health. The highest improvement was for the dimension of overall satisfaction (1.18), followed by sexual desire (1.12), intercourse satisfaction (0.68), and ED (−0.041). While no change was observed for orgasm function. In the same vein, following a randomized placebo-controlled trial, Parandavar et al. (31) reported improved sexual function mean score for those who received melatonin (31). Furthermore, some animal studies also suggested improvements in the sexual function of rats following melatonin administration (33, 34). As mentioned before, opioid addiction causes several mental health issues. In this line, there are evidence regarding the positive effect of melatonin on oxidative stress, which in turn translates into better mental health (35, 36). Our findings indicated the considerable impact of melatonin on investigated dimensions of mental health. Similarly, Shabani et al. (37), who studied the impact of melatonin administration on mental health parameters in women with polycystic ovary syndrome following a randomized, double-blind, placebo-controlled trial, reported positive effects of melatonin on mental health (37). In a clinical randomized trial on those under MMT, Ghaderi et al. (21) reported similar results concerning the impact of melatonin on mental health (21).

zolpidem is an imidazopyridine, a non-benzodiazepine with sedative-hypnotic effects that is widely used to treat mental health issues, mainly due to its high absorption rate. Hence, it can be consumed later in the night without worrying about residual cognitive impairment the next morning (38). In addition, zolpidem is known for its rapid action and low residual and rebound effects. In the present study, zolpidem only was associated with significant improvement in the sexual desire dimension. In addition, its effect was lower than that of melatonin for both investigated outcomes. zolpidem did not cause improved mental health status. While this finding is in line with some studies such as that of Eslami-Sharbabaki et al. (39), it is not in line with several other studies. For instance, Dang et al. (38) reported significant effects on the management of mental health issues in subjects with drug abuse (38). Or in a randomized controlled study, Shakya et al. (40) reported positive effects of 10 mg zolpidem on sleep quality, pain management, and reducing depression (40). This difference can be attributed to factors such as low dose of the drug or short follow-up period of our study. In this study, we examined the effect of 1 month of zolpidem, although various studies have reported side effects such as the risk of suicide, rebound insomnia, falls, hip fractures, etc. in long-term use or with high doses of this drug, and this point should be considered in the administration of zolpidem (41, 42).

According to the best knowledge of the authors, no study has compared the effect of melatonin and zolpidem concerning either sexual function or mental health; hence, we couldn't find comparable findings to mention in this study. Eventually, it should be noted that some evidence indicated a gender difference concerning the effect of zolpidem on sexual function, with higher levels of plasma concentrations in women (43).

In Iran, besides methadone treatment protocol, there is no standard treatment for patients' psychological and sexual problems. Therefore, the results of this study can help addiction therapists to manage their patients' problems.

### Limitations

It is necessary to mention some limitations of our study, including the withdrawal of some participants, short follow up period, intervention only on male gender and being a single center study. Hence, caution should be taken when generalizing the findings.

## Data Availability Statement

The original contributions presented in the study are included in the article/supplementary material, further inquiries can be directed to the corresponding author.

## Ethics Statement

The studies involving human participants were reviewed and approved by the Research Ethics Committee of the Isfahan University of Medical Sciences approved and supported the trial (code IR.MUI.MED.REC.1399.813). The patients/participants provided their written informed consent to participate in this study.

## Author Contributions

All authors listed have made a substantial, direct, and intellectual contribution to the work and approved it for publication.

## Funding

This research benefited from the support of the Isfahan University of Medical Sciences.

## Conflict of Interest

The authors declare that the research was conducted in the absence of any commercial or financial relationships that could be construed as a potential conflict of interest.

## Publisher's Note

All claims expressed in this article are solely those of the authors and do not necessarily represent those of their affiliated organizations, or those of the publisher, the editors and the reviewers. Any product that may be evaluated in this article, or claim that may be made by its manufacturer, is not guaranteed or endorsed by the publisher.

## References

[1] Suleiman D. Mental health disorders in Nigeria: a highly neglected disease. Ann Niger Med. (2016) 10:47–47. 10.4103/0331-3131.206214

[2] Radez J Reardon T Creswell C Lawrence PJ Evdoka-Burton G Waite P. Why do children and adolescents (not) seek and access professional help for their mental health problems? A systematic review of quantitative and qualitative studies. Eur Child Adolesc Psychiatry. (2021) 30:183–211. 10.1007/s00787-019-01469-4 31965309 PMC7932953

[3] Hossain MM Tasnim S Sultana A Faizah F Mazumder H Zou L. Epidemiology of mental health problems in COVID-19: a review. F1000Res. (2020) 9:636. 10.12688/f1000research.24457.1 33093946 PMC7549174

[4] Nochaiwong S Ruengorn C Thavorn K Hutton B Awiphan R Phosuya C. Global prevalence of mental health issues among the general population during the coronavirus disease-2019 pandemic: a systematic review and meta-analysis. Sci Rep. (2021) 11:1–18. 10.1038/s41598-021-89700-8 33986414 PMC8119461

[5] World Drug Report 2021 in Special Points of Interest. United Nations publication (2021).

[6] Substance Abuse Mental Health Services Administration. Key substance use and mental health indicators in the United States: Results from the 2019 National Survey on Drug Use and Health (HHS Publication No.PEP20-07-01-001, NSDUH Series H-55). Rockville, MD: Center for Behavioral Health Statistics and Quality, Substance Abuse and Mental Health Services Administration (2020). Available online at: https://www.samhsa.gov/data/

[7] Jones CM McCance-Katz EF. Co-occurring substance use and mental disorders among adults with opioid use disorder. Drug Alcohol Depend. (2019) 197:78–82. 10.1016/j.drugalcdep.2018.12.030 30784952

[8] Drake RE Mueser KT Brunette MF. Management of persons with co-occurring severe mental illness and substance use disorder: program implications. World Psychiatry. (2007) 6:131. 18188429 PMC2174596

[9] Sullivan MD. Depression effects on long-term prescription opioid use, abuse, and addiction. Clin J Pain. (2018) 34:878–84. 10.1097/AJP.0000000000000603 29505419

[10] Hasler BP Smith LJ Cousins JC Bootzin RR. Circadian rhythms, sleep, and substance abuse. Sleep Med Rev. (2012) 16:67–81. 10.1016/j.smrv.2011.03.004 21620743 PMC3177010

[11] Kurth ME Sharkey KM Millman RP Corso RP Stein MD. Insomnia among methadone-maintained individuals: the feasibility of collecting home polysomnographic recordings. J Addict Dis. (2009) 28:219–25. 10.1080/10550880903014155 20155590 PMC2874989

[12] Carey MG Al-Zaiti SS Dean GE Sessanna L Finnell DS. Sleep problems, depression, substance use, social bonding, and quality of life in professional firefighters. J Occup Environ Med. (2011) 53:928. 10.1097/JOM.0b013e318225898f 21785370 PMC3486736

[13] Gates P Albertella L Copeland J. Cannabis withdrawal and sleep: a systematic review of human studies. Subst Abus. (2016). 37:255–69. 10.1080/08897077.2015.1023484 25893849

[14] Le TA Le MQT Dang AD Dang AK Nguyen CT Pham HQ. Multi-level predictors of psychological problems among methadone maintenance treatment patients in difference types of settings in Vietnam. Subst Abuse Treat Prev Policy. (2019) 14:1–10. 10.1186/s13011-019-0223-4 31533764 PMC6751619

[15] Yen C-N Wang CS-M Wang T-Y Chen H-F Chang H-C. Quality of life and its correlates among heroin users in Taiwan. Kaohsiung J Med Sci. (2011) 27:177–83. 10.1016/j.kjms.2010.09.003 21527184 PMC11916587

[16] Ghadigaonkar DS Murthy P. Sexual dysfunction in persons with substance use disorders. J Psychosexual Health. (2019) 1:117–21. 10.1177/2631831819849365

[17] Mishra SK Srivastava M. Sexual dysfunction in substance abuse and dependence: a cross-sectional survey. ASEAN J Psychiatry. (2018) 19:1–7.

[18] Xie Z Chen F Li WA Geng X Li C Meng X. A review of sleep disorders and melatonin. Neurol Res. (2017) 39:559–65. 10.1080/01616412.2017.1315864 28460563

[19] Farnia V Tatari F Alikhani M Shakeri J Taghizadeh M Karbasizadeh H. Rosa Damascena oil improved sexual function and testosterone in male patients with opium use disorder under methadone maintenance therapy–results from a double-blind, randomized, placebo-controlled clinical trial. Drug Alcohol Depend. (2017) 176:117–25. 10.1016/j.drugalcdep.2017.02.008 28531768

[20] Farnia V Alikhani M Ebrahimi A Golshani S Bahmani DS Brand S. Ginseng treatment improves the sexual side effects of methadone maintenance treatment. Psychiatry Res. (2019) 276:142–50. 10.1016/j.psychres.2019.05.004 31082749

[21] Ghaderi A Banafshe HR Mirhosseini N Motmaen M Mehrzad F Bahmani F. The effects of melatonin supplementation on mental health, metabolic and genetic profiles in patients under methadone maintenance treatment. Addict Biol. (2019) 24:754–64. 10.1111/adb.12650 29949232

[22] Aziriova S Bednarova KR Krajcirovicova K Hrenak J Rajkovicova R Arendasova K. Doxorubicin-induced behavioral disturbances in rats: protective effect of melatonin and captopril. Pharmacol Biochem Behav. (2014) 124:284–9. 10.1016/j.pbb.2014.06.021 24983779

[23] Hansen MV Andersen LT Madsen MT Hageman I Rasmussen LS Bokmand S. Effect of melatonin on depressive symptoms and anxiety in patients undergoing breast cancer surgery: a randomized, double-blind, placebo-controlled trial. Breast Cancer Res Treat. (2014) 145:683–95. 10.1007/s10549-014-2962-2 24756186

[24] Jung M. Zolpidem overdose: a dilemma in mental health. Health Care Manag. (2018) 37:86–9. 10.1097/HCM.0000000000000199 29251649

[25] Jun D Johnston V Kim J-M O'Leary S. Cross-cultural adaptation and validation of the depression, anxiety and stress scale-21 (DASS-21) in the Korean working population. Work. (2018) 59:93–102. 10.3233/WOR-172661 29439379

[26] Rosen RC Cappelleri JC. Gendrano Nr. The International Index of Erectile Function (IIEF): a state-of-the-science review. Int J Impot Res. (2002) 14:226–44. 10.1038/sj.ijir.3900857 12152111

[27] Rajabi G Dastan N Shahbazi M. Reliability and validity of the sexual self-efficacy scale-erectile functioning. Iran J Psychiatry Clin Psychol. (2012) 18:74–82.4078907

[28] Chan Y-Y Yang S-N Lin J-C Chang J-L Lin J-G Lo W-Y. Inflammatory response in heroin addicts undergoing methadone maintenance treatment. Psychiatry Res. (2015) 226:230–4. 10.1016/j.psychres.2014.12.053 25660662

[29] Lahon K Shetty HM Paramel A Sharma G. Sexual dysfunction with the use of antidepressants in a tertiary care mental health setting–a retrospective case series. J Pharmacol Pharmacother. (2011) 2:128. 10.4103/0976-500X.81913 21772780 PMC3127346

[30] Outhoff K. Antidepressant-induced sexual dysfunction: CPD. S Afr Fam Pract. (2009) 51:298–302. 10.1080/20786204.2009.10873868

[31] Parandavar N Abdali K Keshtgar S Emamghoreishi M Amooee S Mosalanejad L. The effect of melatonin on the sexual function among postmenopausal women: a randomized placebo-controlled trial. Nurs Midwifery Stud. (2017) 6:149–55. 10.4103/nms.nms_47_17

[32] Sharifiaghdas F Azadvari M Shakhssalim N Roohi-Gilani K Rezaei-Hemami M. Female sexual dysfunction in type 2 diabetes: a case control study. Med Princ Pract. (2012) 21:554–9. 10.1159/000339118 22739547

[33] Andersson KE. Mechanisms of penile erection and basis for pharmacological treatment of erectile dysfunction. Pharmacol Rev. (2011) 63:811–59. 10.1124/pr.111.004515 21880989

[34] Qiu X-F Li X-X Chen Y Lin H-C Yu W Wang R. Mobilisation of endothelial progenitor cells: one of the possible mechanisms involved in the chronic administration of melatonin preventing erectile dysfunction in diabetic rats. Asian J Androl. (2012) 14:481. 10.1038/aja.2011.161 22367180 PMC3720169

[35] Turek FW Gillette MU. Melatonin, sleep, and circadian rhythms: rationale for development of specific melatonin agonists. Sleep Med. (2004) 5:523–32. 10.1016/j.sleep.2004.07.009 15511698

[36] Pereira N Naufel MF Ribeiro EB Tufik S Hachul H. Influence of dietary sources of melatonin on sleep quality: a review. J Food Sci. (2020) 85:5–13. 10.1111/1750-3841.14952 31856339

[37] Shabani A Foroozanfard F Kavossian E Aghadavod E Ostadmohammadi V Reiter RJ. Effects of melatonin administration on mental health parameters, metabolic and genetic profiles in women with polycystic ovary syndrome: a randomized, double-blind, placebo-controlled trial. J Affect Disord. (2019) 250:51–6. 10.1016/j.jad.2019.02.066 30831541

[38] Dang A Garg A Rataboli PV. Role of zolpidem in the management of insomnia. CNS Neurosci Ther. (2011) 17:387–97. 10.1111/j.1755-5949.2010.00158.x 20553305 PMC6493830

[39] Eslami-Shahrbabaki M Barfeh B Nasirian M. Persistent psychosis after abuse of high dose of zolpidem. Addict Health. (2014) 6:159. 25984284 PMC4354222

[40] Shakya H Wang D Zhou K Luo Z-Y Dahal S Zhou Z-K. Prospective randomized controlled study on improving sleep quality and impact of zolpidem after total hip arthroplasty. J Orthop Surg Res. (2019) 14:1–9. 10.1186/s13018-019-1327-2 31481074 PMC6724364

[41] Cho C-H Jee H-J Nam Y-J An H Kim L Lee H-J. Temporal association between zolpidem medication and the risk of suicide: A 12-year population-based, retrospective cohort study. Sci Rep. (2020) 10:1–8. 10.1038/s41598-020-61694-9 32184423 PMC7078307

[42] Edinoff AN Wu N Ghaffar YT Prejean R Gremillion R Cogburn M. Zolpidem: efficacy and side effects for insomnia. Health Psychol Res. (2021) 9:24927. 10.52965/001c.24927 34746488 PMC8567759

[43] Roehrs TA Roth T. Gender differences in the efficacy and safety of chronic nightly zolpidem. J Clin Sleep Med. (2016) 12:319–25. 10.5664/jcsm.5574 26446253 PMC4773634

